# Correlation Analysis of Attention and Intelligence of Preterm Infants at Preschool Age: A Premature Cohort Study

**DOI:** 10.3390/ijerph20043357

**Published:** 2023-02-14

**Authors:** Wei-Chih Chin, Wei-Chi Wu, Jen-Fu Hsu, I. Tang, Tsung-Chieh Yao, Yu-Shu Huang

**Affiliations:** 1 Department of Child Psychiatry and Sleep Center, Chang Gung Memorial Hospital, Taoyuan 333, Taiwan; 2 College of Medicine, Chang Gung University, Taoyuan 333, Taiwan; 3 Department of Ophthalmology, Chang Gung Memorial Hospital, Taoyuan 333, Taiwan; 4 Division of Pediatric Neonatology, Department of Pediatrics, Chang Gung Memorial Hospital, Taoyuan 333, Taiwan; 5 Department of Pediatrics, Chang Gung Memorial Hospital, Taoyuan 333, Taiwan

**Keywords:** prematurity, neurocognition, preschool age, intelligence, attention

## Abstract

Objective: Developmental delay in neurocognitive function has been reported in premature children. This cohort study prospectively followed preterm infants following birth, and herein we present the four-year longitudinal follow-up data of cognitive development at preschool age and analyze correlated factors. Methods: Term and preterm children received regular clinical evaluations and development assessments after birth, and at age 4 ± 1 years, they received the Wechsler-preschool and primary scale of intelligence, Fourth Edition (WPPSI-IV), excluding those with full-scale intelligence quotient < 70. A total of 150 participants received Conners Kiddie Continuous Performance Test (K-CPT), while 129 participants received ophthalmic evaluation. We adopted Chi-square test, ANOVA, and post hoc analysis to compare group differences. Correlations with K-CPT and WPPSI-IV were analyzed using Pearson’s correlation. Results: Group 1 consisted of 25 full-term children, group 2 had 94 preterm children with birth-weight of ≥ 1500 g, and group 3 had 159 preterm children with birth-weight of < 1500 g. Group 1 was the healthiest group and had the best performance in attention and intelligence, while group 3 had the worst physical condition and cognitive performance. The correlation analysis revealed that perinatal factors, including gestational age, birth weight, Apgar scores, and physical conditions, significantly correlated with WPPSI-IV and K-CPT variables. Gender significantly correlated with object assembly of WPSSI-IV and clinical index of K-CPT. Among vision-related variables, best corrected visual acuity correlated most with K-CPT, including clinical index, Omission, and hit reaction time standard error of K-CPT, as well as significantly correlated with information and bug search of WPPSI-IV. Conclusions: Preterm children at preschool age still had poorer cognitive performance than full-term children, especially those with birth BW less than 1500 g. Gender and vision are correlated with cognitive deficits. Continuous monitoring with comprehensive assessments is recommended.

## 1. Introduction

Prematurity is highly correlated with developmental delays [1,2,3,4]. Such correlation has been found not only in severe neurodevelopmental sequelae, such as cerebral palsy and cognitive deficiency, but also in subtle neuromotor dysfunction, such as difficulties in social contact and learning [5,6,7,8,9]. Despite advances in neonatal care, rapid development of the fetal brain can be disrupted by preterm delivery, leading to cognitive impairments, and studies have demonstrated that alterations in brain structures can be found even in late preterm children, including smaller brain volume, less developed myelinization, and more immature gyros folding [10]. Common postnatal morbidities, such as atrial septum deficit (ASD), ventricular septum deficit (VSD), bronchopulmonary dysplasia (BPD), pulmonary hypertension (PH), patent ductus arteriosus (PDA), respiratory distress syndrome (RDS), retinopathy of prematurity (ROP), intraventricular hemorrhage (IVH), necrotizing enterocolitis (NEC), intraventricular hemorrhage (IVH), periventricular leukomalacia (PVL), pneumonia, and sepsis can also increase the risk of cognitive deficits [11,12,13]. Cognitive deficits of preterm children can result in impaired academic performance at school age and increase the need for both special education and mental health care [14,15,16,17].

Most domains of cognition can be affected in the most severe cases, but minor neurodevelopmental dysfunction can be more prevalent in preterm children [18,19,20]. Up to 25–50% preterm children have minor neurodevelopmental dysfunction problems [21] and may have specific neuropsychological impairments instead of pervasive impairments, and their full-scale intelligence quotient (IQ) can be average. These minor dysfunctions can be evaluated in early childhood [5,9,22,23], and Hsu et al. (2013) have reported minor neurodevelopmental dysfunctions at 6 months corrected age by the Bayley Scales of Infant Development and the Denver Developmental Screening Test [19]. At age two, our cohort study found that these children could have a higher rate of development delay, more sleep problems associated with sleep disordered breathing, and more craniofacial development problems (narrow high palate) [20]. Although the severity of cognitive deficits may be minor, a previous meta-analysis has shown that they can persist as preterm children grow [24], and the impact on later life should not be underestimated. Studies of preterm children at the preschool stage confirmed their vulnerability to cognitive impairment. A recent meta-analysis of neuropsychological functioning in preterm children at preschool age included studies of children born after 1994 and found that they had worse mean IQ, attention, memory, visuomotor integration skill, and executive function when compared with full-term children [24]. In addition to the likelihood of more cognitive impairments in preterm children, a recent cohort study found that the prediction of cognitive risk improves with age, and the assessments at 6 years old offer optimal diagnostic accuracy for development in adolescence (age 12) [25]. This finding highlighted the importance of continuous monitoring. However, the full-scale IQ score can be oversimplified to represent the exact neurocognitive development of preterm children, and the cognitive profiles of preterm children can have large heterogeneity in different cognitive subscales [26]. Nearly half of preterm children with cognitive difficulties at school age were found to have normal development when aged 1 to 3 years [27]. Therefore, simplified assessments may overlook impairments and delay intervention, thus indicating that more comprehensive assessments be provided.

In addition to monitoring the cognition of preterm children, analysis and identification of correlated factors can help identify high-risk children and provide early intervention resources. In our previous studies, we found that gestational age and birth body weight played important roles in the risk of these dysfunctions [19]. Likewise, previous studies have also reported that birth weight or gestational age were associated with intelligence [28,29,30,31] and attention deficits [28,32,33,34], Other perinatal and postnatal factors can also correlate with children’s cognitive development.

The assessment of neurocognitive development in the preschool stage can be valuable since neuropsychological assessments, such as attention and intelligence tests, are available at this time to increase the understanding of preterm children’s cognition [24], and early intervention can decrease adverse outcomes. However, studies during this age period often adopt less comprehensive assessments, for example, the revised version of WPPSI rather than WPPSI-IV, because of time and resource constraints, which means that not all subtests are evaluated. The study designs have often been retrospective or cross-sectional, and the follow-up period in studies with prospective designs has been short. Our cohort study has already published our 6-month and 2-year follow-up data [19,20], and in this study, we evaluated both attention and intelligence of preterm children by WPPSI-IV and Conners Kiddie Continuous Performance Test (K-CPT), aiming at more comprehensive neurocognitive evaluation. Furthermore, since ROP is prevalent in preterm children and most cognitive tasks require vision, ophthalmic assessment has also been performed in this study. We presented the fourth year longitudinal follow-up data of the neurocognitive development of preterm children after they entered preschool age (age 4 ± 1) and analyzed the correlated factors.

## 2. Materials and Methods

### 2.1. Study Population

This cohort study of preterm children was approved by the institutional review board of Chang Gung Memorial Hospital (201801537A3). Women delivering in our hospital since 2010 have been invited if their children met the inclusion criteria without any exclusion criteria. Their children were enrolled in the cohort study with their parents’ informed consent.

#### 2.1.1. Inclusion Criteria

(1) Term infants born between 37 and 40 gestational weeks with birth weight of more than 2500 g whose parents were willing to sign the informed consent (group 1, the control group).
(2) All infants born before 37 gestational weeks whose parents were willing to sign the informed consent. According to their birth weight, these infants were divided into group 2 (≥1500 g) and group 3 (<1500 g).
(3) Children who were able to complete neurocognitive function tests and had a full-scale intellectual quotient more than 70.
(4) Children whose parents were willing and able to complete questionnaires.

#### 2.1.2. Exclusion Criteria

(1) Since the study aimed to investigate minor neurodevelopmental dysfunction, we had to exclude neonates with severe physical impairments due to perinatal injures, severe hypoxic ischemic encephalopathy, severe congenital malformations, confirmed chromosome anomalies, and the requirement of long-term intubation.
(2) Neonates who were unable to receive the systemic regular follow-up required by this cohort study.
(3) Children who were unable to cooperate with neurocognitive function tests or who had a full-scale intellectual quotient less than 70.
(4) Children whose parents were unwilling or unable to complete questionnaires.

### 2.2. Procedure

Demographic and clinical data, including obstetric and birth data, were collected for all term and preterm infants following enrollment. Obstetric and birth data included gestational age (GA), birth weight (BW), and Apgar score. Other clinical data included physical diseases (atrial septum deficit, ASD; bronchopulmonary dysplasia, BPD; necrotizing enterocolitis, NEC; intraventricular hemorrhage, IVH; patent ductus arteriosus, PDA; periventricular leukomalacia, PVL; respiratory distress syndrome, RDS; ventricular septum deficit, VSD; anemia; sepsis) and blood transfusion and surfactant use after birth.

**Figure 1:**
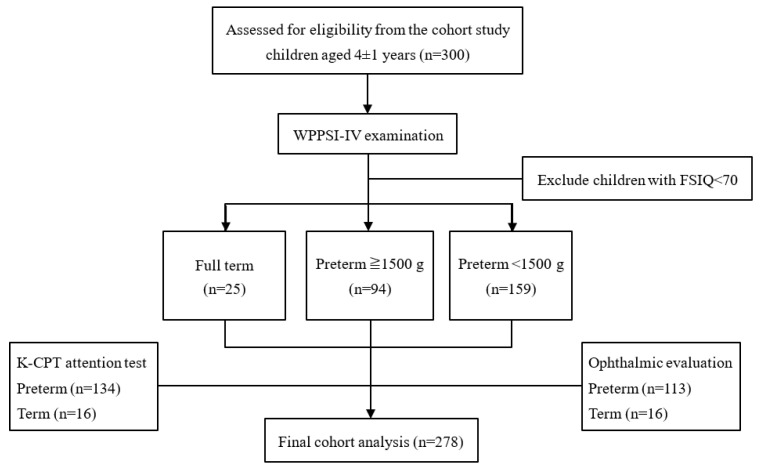
Flow chart of the study protocol. WPPSI: the Wechsler preschool and primary scale of intelligence, Fourth Edition; FSIQ: full-scale intelligence quotient; K-CPT: Conners Kiddie Continuous Performance Test.

Every 6 months, all participants received a clinical evaluation by pediatric ear–nose–throat and oro-maxillo-facial and developmental specialists and a development assessment by the Denver Developmental Screening Test—second edition. The Bailey Scale of Infant Development was performed by child psychologists. The results of the initial 2-year follow-up have already been published [20]. When children were at age 4 ± 1 year, all children received cognitive function evaluation using the Chinese version of the Wechsler preschool and primary scale of intelligence, Fourth Edition (WPPSI-IV) and Child Behavior Checklist. Furthermore, 150 participants (134 preterm children and 16 term children) received K-CPT to evaluate their attention, and 129 participants (113 preterm children with retinopathy of prematurity and 16 term children) also received ophthalmic evaluation (Figure 1).

### 2.3. Intelligence Evaluation

We evaluated participants’ cognitive function using the Wechsler preschool and primary scale of intelligence, Fourth Edition (WPPSI-IV, Chinese Behavioral Science CO., Taipei, Taiwan) [35], performed by certified and experienced pediatric psychiatrists and psychologists. The original version of WPPSI-IV was previously validated with excellent internal consistency. The reliability coefficient is 0.96, and the test–retest correction is 0.93 [36]. The Chinese version validated in Taiwan also shows good internal consistency with a reliability coefficient of 0.86–0.96 and a test–retest correlation of 0.72–0.89 [35]. The test has two age ranges, with the younger group ranging from 2 years 6 months to 3 years 11 months, and the older group ranging from 4 years to 7 years 7 months. Eight and fifteen subtests consist of the younger and older range tests, respectively. The raw scores of subtests are converted to age-corrected standard scaled scores. These age-corrected scores are further calculated into three primary index scales of the younger range test, verbal comprehension index (VCI), visual spatial index (VSI), and working memory index (WMI), and five primary index scales of the older range test, VCI, VSI, WMI, fluid reasoning index (FRI), and processing speed index (PSI). These primary index scales were further combined into the full-scale intelligence quotient (FSIQ) score as the overall intelligence of each participant.

### 2.4. Attention Evaluation

We evaluated participants’ attention using the Conners Kiddie Continuous Performance Test (K-CPT). K-CPT is a computerized visual task that measures attention, vigilance, and impulsivity of preschool children aged 4–6 years [37]. A series of pictures appear on the screen, and children have to respond by pressing the space bar. When a target stimulus is presented, the children should press the key. When a non-target stimulus is presented, the children should not respond. It takes 7.5 min to complete and measures children’s response time, omission errors (fail to respond to target), commission errors (response to non-target), change in reaction time speed, and consistency. Children’s performance in K-CPT generates T scores that include omission errors, commission errors, hit reaction time (Hit RT), hit RT standard error (Hit RT SE), variability, detectability, response style, perseverations, Hit RT block change, Hit SE block change, Hit RT inter-stimulus interval (ISI) change, Hit SE ISI change, and the overall clinical index (CI). K-CPT was administered by experienced child psychotherapists in this study.

### 2.5. Ophthalmic Evaluation

Participants with ROP received an ophthalmic examination that included slit-lamp biomicroscopy, indirect ophthalmoscopy, and uncorrected and best corrected visual acuity (BCVA) assessments. We used an automatic kerato-refractometer (KR-8100A; Topcon, Tokyo, Japan) with manual refraction for the cycloplegic refraction to measure participants’ spherical power (SPH), cylinder (CYL), and spherical equivalent (SE: SPH + CYL/2).

### 2.6. Statistical Analysis

Continuous variables were presented by means and standard deviations, while categorical variables were calculated into frequencies and percentages. To compare demographic and clinical data, WPPSI-IV, and K-CPT between groups, data were analyzed using Chi-square test, ANOVA, and post hoc analysis. The correlations of demographic and clinical data and WPPSI-IV and K-CPT were analyzed by Pearson’s correlation with Bonferroni correction. Statistical significance was defined as *p* < 0.05. SPSS version 18 was used for all statistical analyses.

## 3. Results

We included a total of 278 participants for the final cohort analysis after removing 22 children with FSIQ less than 70. Group 1 consisted of 25 full term children, group 2 had 94 preterm children with birth weight of ≥ 1500 g, and group 3 included 159 preterm children with birth weight of < 1500 g. Their demographic and clinical data are presented in Table 1. Group 1 was the healthiest group of the three groups, had significant higher GA (*p* < 0.001, 1 > 2 > 3), higher BW (*p* < 0.001, 1 > 2 > 3), less RDS (*p* < 0.001, 3 > 2 > 1), less anemia (*p* < 0.001, 3 > 2 > 1), and received fewer blood transfusions (*p* < 0.001, 3 > 2 > 1) than groups 2 and 3. Group 3 had the worst physical condition and had significant lower GA (*p* < 0.001, 1 > 2 > 3), lower BW (*p* < 0.001, 1 > 2 > 3), lower Apgar score at both 1 min and 5 min (*p* < 0.001, 1,2 > 3), more physical diseases, including ASD (*p* < 0.001, 3 > 1,2), BPD (*p* < 0.001, 3 > 1,2), pneumonia (*p* < 0.001, 3 > 1,2), PDA (*p* < 0.001, 3 > 1,2), RDS (*p* < 0.001, 3 > 2 > 1), NEC (*p* < 0.001, 3 > 2), IVH (*p* < 0.001, 3 > 1,2), ROP (*p* < 0.001, 3 > 1,2), anemia (*p* < 0.001, 3 > 2 > 1) and sepsis (*p* < 0.001, 3 > 1,2), as well as more blood transfusions (*p* < 0.001, 3 > 2 > 1) and surfactant use (*p* < 0.001, 3 > 1,2) after birth.

Table 2 shows the comparison of K-CPT variables between groups after adjusting for age, GA, BPD, and IVH. Group 3 had the worst performance in Omission (*p* < 0.001, 3 > 1,2), Hit RT (*p* = 0.035, 3 > 2), Hit RT SE (*p* = 0.002, 3 > 1,2), variability (*p* = 0.029, 3 > 2), and Hit SE ISI change (*p* = 0.003, 3 > 2).

Table 3 shows the comparison of WPPSI-IV variables between groups after adjusting for age, GA, BPD, and IVH. Group 1 had the best cognitive performance, while group 3 had the worst performance in WPPSI-IV variables, including full-scale intelligence quotient (FSIQ) (*p* < 0.001, 1,2 > 3), all five index scores, VCI (*p* = 0.004, 1 > 3), VSI (*p* < 0.001, 1,2 > 3), FRI (*p* < 0.001, 1,2 > 3), WMI (*p* < 0.001, 1,2 > 3), PSI (*p* < 0.001, 1,2 > 3), and almost all subtests, as well as (SI) (*p* = 0.001, 1,2 > 3), information (IN) (*p* = 0.002, 1 > 3), object assembly (OA) (*p* < 0.001, 1,2 > 3), block design (BD) (*p* < 0.001, 1,2 > 3), picture concept (PC) (*p* < 0.001, 1,2 > 3), matric reasoning (MR) (*p* = 0.001, 1 > 3), zoo locations (ZL) (*p* < 0.001, 1,2 > 3), picture memory (PM) (*p* < 0.001, 1,2 > 3), and bug search (BS) (*p* < 0.001, 1,2 > 3). The only exception was the subtest score for cancellation (CA) (*p* = 0.023), which showed no group difference.

We further analyzed the correlations of K-CPT and WPPSI-IV variables and demographic and perinatal factors, as well as vision at around age 4. The results are shown in Table 4 (K-CPT) and Table 5 (WPPSI-IV).

- Correlations with K-CPT (Table 4):

Gender was significantly correlated with CI of K-CPT. Among perinatal factors, GA was significantly correlated with CI, Omission, Hit RT, Hit RT SE, and Hit SE ISI change. BW was significantly correlated with Omission, Hit RT SE, and Hit SE ISI change, while gender was significantly correlated with CI. Apgar score 1 min after birth was significantly correlated with CI, Omission, Hit RT, Hit RT SE, variability. Furthermore, Apgar score 5 min after birth was significantly correlated with Omission, Hit RT SE, and variability.

Significant correlations were found between physical diseases and K-CPT variables. RDS was significantly correlated with Omission, Hit RT SE, and Hit SE ISI change. BPD was significantly correlated with CI, Omission, Hit RT, Hit RT SE, and variability. ROP was significantly correlated with CI, Omission, Hit RT, Hit RT SE, and variability. Pneumonia was significantly correlated with CI, Omission, Hit RT SE, and Hit RT ISI change. ASD was significantly correlated with detectability and response style, and NEC was significantly correlated with Hit RT SE, variability, and detectability. VSD was significantly correlated with perseveration, and anemia was significantly correlated with Omission. Blood transfusion was significantly correlated with Omission, Hit RT SE, and Hit SE ISI change, and surfactant use was significantly correlated with CI, Omission, Hit RT, and Hit RT SE.

With Bonferroni correction (*p* < 0.00015), correlations with K-CPT variables were less. GA was significantly correlated with Omissions and Hit RT SE. Apgar score 1 min and 5 min after birth were correlated with Omission, Hit RT SE, and variability. ROP was correlated with CI, Omission, and Hit RT SE. BPD was correlated with Omission and Hit RT SE. Pneumonia was correlated with Omission. Surfactant use was correlated with Omission and Hit RT SE.

- Correlations with WPPSI-IV (Table 5):

Current age was significantly correlated with SI of WPPSI-IV, and gender was significantly correlated with OA of WPPSI-IV. Among perinatal factors, GA and BW were significantly correlated with all WPPSI-IV variables. Apgar score 1 min after birth was significantly correlated with all WPPSI-IV variables except IN, SI, and CA. Meanwhile, Apgar score 5 min after birth was also significantly correlated with many WPPSI-IV variables, including FSIQ, VSI, FRI, WMI, BD, BS, PM, PC, ZL, and OA. RDS, BPD, and ROP were significantly correlated with all WPPSI-IV variables. Pneumonia was significantly correlated with all WPPSI-IV variables except PSI. Sepsis was significantly correlated with all WPPSI-IV variables except VCI, PSI, and BS. Anemia was significantly correlated with all WPPSI-IV variables except FRI, BD, and CA. PDA was significantly correlated with FSIQ, VSI, WMI, BD, IN, BS, SI, PC, ZL, and OA. IVH was significantly correlated with FSIQ, VSI, WMI, BD, and OA. NEC was significantly correlated with BS and PC. Blood transfusion and surfactant use after birth were significantly correlated with all WPPSI-IV variables.

With Bonferroni correction (*p* < 0.00013), correlations with WPPSI-IV variables were also fewer. GA was correlated with all WPPSI-IV variables except VCI, IN and CA. BW was correlated with all WPPSI-IV variables except VCI, MR and CA. Apgar score 1 min after birth was correlated with FSIQ, VSI, WMI, BD, ZL, and OA, and Apgar score 5 min after birth was correlated with FSIQ, BD, and ZL. PDA was correlated with WMI, ZL, and OA. ROP was correlated with all WPPSI-IV variables except VCI, IN and SI. RDS was correlated with all WPPSI-IV variables except VCI, IN, SI, PC and CA. BPD was correlated with all WPPSI-IV variables except VCI and CA. Sepsis was correlated with FSIQ, VSI, WMI, ZL, and OA. Pneumonia was correlated with FSIQ, VSI, FRI, WMI, BD, SI, ZL, and OA. Anemia was correlated with FSIQ, WMI, PM, and ZL. Blood transfusion was correlated with FSIQ, WMI, PM, ZL, and OA. Surfactant us was correlated with FSIQ, VSI, FRI, WMI, PSI, BD, PC, ZL, and OA.

- Correlation between K-CPT, WPPSI-IV, and vision at around age four (Table 4 and Table 5):

Although ROP as a perinatal factor was significantly correlated with variables of K-CPT and WPPSI-IV, only BCVA of the ophthalmic assessment was significantly correlated with IN and BS of WPPSI-IV (Table 5). We also observed correlations between BCVA and K-CPT variables, including CI, Omission, and Hit RT SE of K-CPT (*p* < 0.05) (Table 4). However, these correlations were non-significant with Bonferroni correction.

## 4. Discussion

Various functions can be assessed for children’s neurocognitive development, and attention and intelligence in particular play important roles in children’s learning. We investigated these two important fields of neurocognition in preterm children. FSIQ, using the revised version of intelligence assessment, may not be representative enough for the overall cognition of preterm children [26]. Therefore, our cohort study prospectively used both K-CPT and WPSSI-IV, rather than the revised WPPSI, to evaluate the cognitive development of preschool children born preterm. Considering the challenges in performing assessment of preschool children, we excluded those who had an FSIQ below 70 to decrease any bias related to difficulties in cooperation. Only 22 children were excluded, accounting for a relatively small percentage of our participants. Although the assessments of this study can be comprehensive, it still had limitations. First, we did not analyze the impact of social and environmental factors on children’s cognitive development, such as mother’s education level or household income, which have been reported to relate to children’s cognition [28]. Second, not all participants received K-CPT and the ophthalmic evaluation. Only preterm children with ROP (N = 103), 10 preterm children and 16 term children, received the ophthalmological exam because of extra time and visit of the examination, as well as concerns about mydriatic use. Additional studies will be needed to confirm our findings in the future. Third, referral bias can be possible with only 25 term children. WPPSI-IV is a norm referenced test which can reduce bias. Poorer cognitive performance of preterm children is a result of relatively ranking instead of absolute performance. Furthermore, because this is a cohort study which represents the real-world clinical condition, treatment factors, such as treatment type, frequency, and duration, cannot be controlled. Thus, differences in the received early intervention can have impact on cognitive development of our preterm children.

Although preterm children with FSIQ lower than 70 were excluded, our study still confirmed the negative cognitive impacts of prematurity on neurocognitive function after adjusting for age, GA, BPD, and IVH. Full-term children had the best cognitive performance (FSIQ = 103.36 ± 16.37), and premature children with birth BW less than 1500 g had the worst performance in FSIQ (FSIQ = 88.74 ± 15.46) and all index scores and subtests, except for cancellation (CA). CA is one of the subtests of PSI, and previous studies have found that the major cognitive deficits of preterm children are perceptual abilities and working memory [26,38,39], but processing speed can be relatively spared. However, these children have heterogeneous cognitive deficits. We should still note that CA is poorer in preterm children with birth BW less than 1500 g when compared to term children, and a recent study has also reported impaired processing speed in preterm children at school age, besides attention and working memory [40].

The finding of relatively spared CA does not indicate that the processing speed should be of lesser concern, and our findings of K-CPT showed that preterm children had difficulties correctly responding to stimuli and sustaining a consistent response speed and also confirmed that preterm children, especially children with birth BW less than 1500 g, can have poorer attention than full-term children. It is not surprising for children with extreme preterm and extreme low birth BW to have poor performance in CPT [41], and children with extremely low BW are more likely to fail the task [42]. Although birth BW has been proven to be correlated with attention deficits in term children, its role in preterm children is less clear. Since earlier gestational age is correlated with lower BW [34,43], lower BW can have no independent effect on attention. After controlling for gestational age, our results of significantly poorer K-CPT performance of children with birth BW less than 1500 g supported that BW may not be a proxy for gestational age. In addition, late-preterm children can also have worse CPT performance than term children [42]. ADHD is highly prevalent in preterm children and may persist into adulthood [44,45,46]. Considering the negative consequences of impaired attention and complex components of cognition, K-CPT is simple to perform and can serve as a good objective tool for the screening and early identification of cognitive deficits in preterm children.

Female gender was significantly and positively correlated with OA, a subtest of visual spatial index, and CI of K-CPT, indicating the association of female gender and better performance. ADHD is more prevalent in male gender, and male disadvantages in the neurodevelopmental outcomes of preterm children have already been confirmed by previous studies [47,48,49,50], which can account for the correlation of better OA and K-CPT performance in female gender. However, these correlations related to gender were less significant with Bonferroni correction. A recent review reported the analysis of gender differences in a small sample of preterm children. They unexpectedly found that female gender can have poorer visuospatial performance [21]. Studies of visual spatial and visuoperceptual function of gender difference in preterm children is currently lacking but is worth further study.

The correlation analysis of perinatal variables confirmed that GA and BW are significantly correlated with preterm children’s cognition in all WPPSI-IV variables, as well as K-CPT performance, even with Bonferroni correction. We also found that Apgar score at both 1 and 5 min and physical diseases after birth were significantly correlated with variables of WPPSI-IV and K-CPT. That is, perinatal factors play important roles in the cognitive development of preterm children despite advances in neonatal care. Discrete but significant cognitive deficits can still be found even if the fetal maturation is interrupted at 34 to 36 gestational weeks [42,51]. Although early detection and treatment of developmental delay is crucial for preterm children with cognitive deficits, the prevention of premature birth remains better than such cures, and the impacts of perinatal factors highlight the importance of routine pregnancy check-up and examinations, especially in high-risk pregnant females.

Early visual impairments can impact the development of cognition, as supported by the findings of significant correlations of ROP with WPPSI-IV and K-CPT variables. We further found that BCVA at around 4 years of age was negatively correlated with IN, a subtest of verbal comprehension, and BS, a subtest of processing speed. BS requires children to discriminate pictures, while IN evaluates not only verbal ability but also children’s general knowledge, and both subtests can be influenced by visual acuity. Correlations between BCVA and CI, Omission, and hit reaction time standard error of K-CPT were also noted, demonstrating the influence of current vision on children’s cognitive performance. However, the correlation was not observed in other WPPSI-IV variables, especially visuospatial domains, such as VSI and related subtests. Although ROP was significantly correlated with several K-CPT variables, we only observed significant correlations between BCVA and K-CPT, and the correlations were not significant with Bonferroni correction. The negative correlation between vision, VSI, and other WPSSI-IV and K-CPT variables may relate to our study’s sample size. Although 113 preterm children had ROP, less than half of our participants received the ophthalmic evaluation. Studies investigating vision and cognitive outcomes in preterm children are still lacking, and studies with larger sample sizes are need.

## 5. Conclusions

In conclusion, FSIQ as a global cognitive outcome may not predate developmental delay, and as comprehensive assessments as possible are needed for those with minor cognitive deficits. We found that preterm children at preschool age still had poorer cognitive performance than full-term children in attention and intelligence, especially those with birth BW less than 1500 g. Prenatal, perinatal, and postnatal factors, including gender, GA, birth BW, physical diseases, and current vision play important roles in the cognitive development of preterm children. Although the diagnostic accuracy of cognitive impairment at school age can become more optimal with age, interventions should be provided early during preterm children’s development, and thus continuous monitoring with comprehensive assessments is recommended, especially in those at higher risk.

## Figures and Tables

**Table 1:** Demographic and clinical data of all participants (*n* = 278).

(*n* = 278)	Group 1 Full-Term (*n* = 25)	Group 2 Preterm ≥ 1500 g (*n* = 94)	Group 3 Preterm < 1500 g (*n* = 159)	*p*-Value	Post Hoc (Scheffe; Bonferroni)
Current age (yr.)	4.09 ± 1.04	4.09 ± 1.06	4.14 ± 0.92	0.936	-
Male, *n* (%)	17 (68.0%)	53 (56.4%)	89 (56.0%)	0.440	-
Perinatal factors:					
GA (wk.)	38.35 ± 2.43	33.52 ± 1.95	27.90 ± 2.77	<0.001 *	1 > 2 > 3
BW (g)	3210.21 ± 442.30	2025.63 ± 453.48	970.81 ± 266.44	<0.001 *	1 > 2 > 3
Apgar score, 1 min	8.6 ± 0.58	7.80 ± 1.51	5.47 ± 2.01	<0.001 *	1,2 > 3
Apgar score, 5 min	9.6 ± 0.58	9.11 ± 1.29	7.60 ± 1.60	<0.001 *	1,2 > 3
ASD	1 (4.0%)	11 (11.7%)	45 (28.3%)	0.001 *	3 > 1,2
VSD	0 (0.0%)	2 (2.1%)	1 (0.6%)	0.462	-
BPD	0 (0.0%)	4 (4.3%)	114 (71.7%)	<0.001 *	3 > 1,2
Pneumonia	0 (0.0%)	5 (5.3%)	65 (40.9%)	<0.001 *	3 > 1,2
PH	0 (0.0%)	2 (2.1%)	17 (10.7%)	0.012 *	3 > 2
PDA	0 (0.0%)	10 (10.6%)	87 (54.7%)	<0.001 *	3 > 1,2
RDS	0 (0.0%)	35 (37.2%)	151 (95.0%)	<0.001 *	3 > 2 > 1
NEC	0 (0.0%)	2 (2.1%)	19 (11.9%)	0.006 *	3 > 2
IVH	0 (0.0%)	6 (6.4%)	54 (34.0%)	<0.001 *	3 > 1,2
ROP	0 (0.0%)	10 (10.6%)	99 (62.3%)	<0.001 *	3 > 1,2
PVL	0 (0.0%)	0 (0.0%)	2 (1.3%)	0.471	-
Anemia	0 (0.0%)	38 (40.4%)	151 (95.0%)	<0.001 *	3 > 2 > 1
Sepsis	1 (4.0%)	9 (9.6%)	74 (46.5%)	<0.001 *	3 > 1,2
Blood transfusion	0 (0.0%)	29 (30.9%)	152 (95.6%)	<0.001 *	3 > 2>1
Surfactant use	0 (0.0%)	7 (7.5%)	99 (62.7%)	<0.001 *	3 > 1,2

* *p*-value < 0.05; *p*-values were calculated using ANOVA test; SD, standard deviation; group 1: full-term, group 2: preterm ≥ 1500 g, group 3: preterm < 1500 g; yr.: year; wk.: weeks; g: gram; GA: gestational age; BW: birth body weight; ASD: atrial septum deficit; VSD: ventricular septum deficit; BPD: bronchopulmonary dysplasia; PH: pulmonary hypertension; PDA: patent ductus arteriosus; RDS: respiratory distress syndrome; NEC: necrotizing enteritis; IVH: intraventricular hemorrhage; ROP: retinopathy of prematurity; PVL: periventricular leukomalacia.

**Table 2:** Comparison of K-CPT variables between groups after adjusting for age, GA, BPD, and IVH.

(*n* = 150)	Group 1 Full-Term (*n* = 16)	Group 2 Preterm ≥ 1500 g (*n* = 72)	Group 3 Preterm < 1500 g (*n* = 62)	*p*-Value	Post Hoc (Bonferroni)
Clinical index	42.54 ± 16.2	46.02 ± 17.76	51.90 ± 19.37	0.083	-
Omissions	44.90 ± 7.03	45.17 ± 7.48	52.30 ± 12.45	0.000 *	3 > 1,2
Commissions	46.81 ± 7.93	49.75 ± 12.22	48.54 ± 8.97	0.560	-
Hit R	55.53 ± 11.02	53.46 ± 11.67	58.32 ± 9.38	0.035 *	3 > 2
Hit RT SE	48.15 ± 10.89	49.15 ± 9.04	54.83 ± 10.02	0.002 *	3 > 2,1
Variability	47.87 ± 10.72	47.32 ± 8.68	51.83 ± 11.01	0.029 *	3 > 2
Detectability	46.17 ± 9.15	47.62 ± 11.80	48.82 ± 10.44	0.647	-
Response Style	50.85 ± 15.97	52.13 ± 15.69	57.45 ± 17.66	0.128	-
Perseverations	52.75 ± 14.67	54.41 ± 16.89	55.02 ± 16.36	0.885	-
Hit RT Block Change	49.20 ± 6.07	45.45 ± 9.94	126.77 ± 625.65	0.484	-
Hit SE Block Change	45.64 ± 7.49	45.12 ± 8.72	47.83 ± 8.73	0.186	-
Hit RT ISI Change	49.90 ± 10.29	51.33 ± 11.67	64.75 ± 70.07	0.202	-
Hit SE ISI Change	46.90 ± 10.23	45.27 ± 8.20	50.26 ± 7.63	0.003 *	3 > 2

* *p*-value < 0.05; *p*-values were calculated using ANOVA test; SD, standard deviation; group 1: full-term, group 2: preterm ≥ 1500 g, group 3: preterm < 1500 g; K-CPT: the Conners Kiddie Continuous Performance; GA: gestational age; BPD: bronchopulmonary dysplasia; IVH: intraventricular hemorrhage; RT: reaction time, SE: standard error, ISI: inter-stimulus interval. Higher values of K-CPT indicate poorer performances.

**Table 3:** Comparison of WPPSI-IV variables between groups after adjusting for age, GA, BPD, and IVH.

(*n* = 278)	Group 1 Full-Term (*n* = 25)	Group 2 Preterm ≥ 1500 g (*n* = 94)	Group 3 Preterm < 1500 g (*n* = 159)	*p*-Value	Post Hoc (Bonferroni)
FSIQ	103.36 ± 16.37	96.92 ± 13.87	88.74 ± 15.46	<0.001 *	1,2 > 3
Index scores					
VCI	105.24 ± 14.78	99.64 ± 15.00	95.04 ± 17.59	0.007 *	1 > 3
VSI	102.00 ± 17.98	98.89 ± 14.70	89.67 ± 16.53	<0.001 *	1,2 > 3
FRI	106.69 ± 15.17	99.48 ± 15.11	88.60 ± 14.74	<0.001 *	1,2 > 3
WMI	98.36 ± 16.34	97.28 ± 13.19	87.22 ± 14.30	<0.001 *	1,2 > 3
PSI	102.15 ± 13.10	92.00 ± 18.92	82.88 ± 17.37	<0.001 *	1,2 > 3
Subtest scores					
SI	11.54 ± 1.05	10.47 ± 2.48	9.01 ± 2.93	0.001 *	1,2 > 3
IN	10.88 ± 2.79	9.65 ± 2.70	8.84 ± 2.99	0.002 *	1 > 3
OA	10.68 ± 3.28	9.68 ± 2.98	8.03 ± 3.26	<0.001 *	1,2 > 3
BD	9.96 ± 3.61	9.85 ± 3.24	7.93 ± 3.36	<0.001 *	1,2 > 3
PC	10.62 ± 3.20	10.40 ± 3.11	7.74 ± 3.08	<0.001 *	1,2 > 3
MR	10.71 ± 2.63	9.40 ± 2.78	8.59 ± 3.03	0.002 *	1 > 3
ZL	9.44 ± 2.90	10.00 ± 2.43	7.82 ± 2.64	<0.001 *	1,2 > 3
PM	9.96 ± 3.58	9.13 ± 2.83	7.72 ± 3.00	<0.001 *	1,2 > 3
CA	9.62 ± 3.23	8.98 ± 2.40	7.76 ± 2.94	0.023 *	-
BS	11.08 ± 2.43	8.61 ± 3.29	6.60 ± 3.52	<0.001 *	1,2 > 3

* *p*-value < 0.05; *p*-values were calculated using ANOVA test; SD, standard deviation; group 1: full-term, group 2: preterm ≥ 1500 g, group 3: preterm < 1500 g; WPPSI: the Wechsler preschool and primary scale of intelligence; GA, gestational age; BPD: bronchopulmonary dysplasia; IVH: intraventricular hemorrhage; FSIQ: full-scale intelligence quotient; VCI: verbal comprehension index; VSI: visual spatial index; FRI: fluid reasoning index; WMI: working memory index; PSI: processing speed index; SI: similarity; IN: information; OA: object assembly; BD: block design; PC: picture concept; MR: matric reasoning; ZL: zoo locations; PM: picture memory; CA: cancellation; BS: bug search.

**Table 4:** The correlations between K-CPT variables demographic data, clinical data, and vision.

	Clinical Index	Omissions	Commissions	Hit RT	Hit RT SE	Variability	Detectability	Response Style	Perseverations	Hit RT Block Change	Hit SE Block Change	Hit RT ISI Change	Hit SE ISI Change
Current age (yr.)	−0.135	0.099	−0.048	0.002	0.076	0.075	0.017	−0.125	0.069	−0.053	0.120	0.146	0.156
Gender	−0.284 **	0.036	0.155	−0.038	0.033	−0.029	0.208 *	0.058	0.081	−0.078	0.063	−0.056	−0.045
Perinatal factors													
GA (wk.)	−0.221 **	−0.353 **	0.060	−0.263 **	−0.319 **	−0.212 *	−0.049	−0.112	−0.029	−0.083	−0.060	−0.210 *	−0.231 **
BW (g)	−0.105	−0.301 **	0.035	−0.185 *	−0.239 **	−0.159	−0.080	−0.114	0.017	−0.069	−0.039	−0.150	−0.259 **
Apgar score, 1 min	−0.269 **	−0.401 **	−0.079	−0.230 **	−0.412 **	−0.331 **	−0.168	0.053	−0.136	−0.130	−0.022	−0.103	−0.089
Apgar score, 5 min	−0.219 *	−0.381 **	−0.063	−0.218 *	−0.387 **	−0.335 **	−0.138	0.030	−0.119	−0.145	0.007	−0.102	−0.059
PH	0.061	0.143	−0.092	0.151	0.066	−0.002	−0.132	0.129	0.038	−0.018	0.020	0.010	−0.032
VSD	−0.024	−0.075	0.181 *	0.037	0.083	0.042	0.085	−0.090	0.298 **	−0.012	−0.029	0.014	0.016
ASD	−0.050	−0.019	−0.206 *	0.122	−0.106	−0.116	−0.229 **	0.239 **	−0.152	−0.037	0.046	0.168	−0.165
PDA	0.135	0.155	0.054	0.172 *	0.200 *	0.068	0.030	0.100	0.125	0.152	0.024	0.000	−0.009
IVH	−0.011	0.037	−0.035	0.027	0.013	0.034	−0.028	−0.001	0.033	0.200 *	0.093	−0.031	0.046
ROP	0.372 **	0.438 **	0.042	0.292 **	0.411 **	0.229 **	0.173 *	−0.053	0.134	0.189 *	0.098	0.030	0.139
RDS	0.147	0.258 **	−0.061	0.129	0.256 **	0.217 *	−0.019	0.128	0.046	0.075	0.014	0.105	0.220 **
NEC	0.137	0.178 *	0.216 *	−0.053	0.221 **	0.224 **	0.252 **	−0.099	0.190 *	−0.024	−0.037	0.008	0.120
BPD	0.278 **	0.382 **	−0.008	0.275 **	0.327 **	0.236 **	0.110	0.132	0.057	0.133	0.112	0.185 *	0.173 *
Sepsis	0.046	0.120	0.059	0.052	0.109	0.026	0.082	−0.019	0.028	0.158	−0.045	0.151	0.024
Pneumonia	0.262 **	0.354 **	0.064	0.175 *	0.299 **	0.157	0.125	0.026	0.165	−0.036	0.058	0.238 **	0.198 *
Anemia	0.088	0.232 **	−0.053	0.155	0.197 *	0.147	0.007	0.173 *	−0.076	0.068	−0.086	0.092	0.163
Blood transfusion	0.141	0.249 **	−0.084	0.215 *	0.249 **	0.210 *	−0.051	0.173 *	0.017	0.070	−0.057	0.112	0.228 **
Surfactant use	0.237 **	0.393 **	−0.009	0.247 **	0.325 **	0.181 *	0.072	0.023	0.063	0.143	0.111	0.187 *	0.165
PVL	0.004	−0.069	0.146	−0.041	0.009	0.036	0.088	−0.056	−0.006	−0.008	−0.021	0.002	0.098
Vision													
BCVA	0.289 *	0.254 *	0.137	0.211	0.255 *	0.126	0.147	0.106	0.139	−0.166	−0.110	−0.065	0.092
SPH	0.048	−0.114	0.141	0.029	−0.083	−0.045	0.170	−0.007	−0.041	0.043	0.251 *	0.014	−0.182
CYL	0.104	0.123	0.115	0.053	−0.003	−0.068	0.120	0.033	0.103	−0.132	−0.129	−0.053	−0.269
SE	0.116	0.083	0.165	0.064	−0.024	−0.077	0.183	0.029	0.091	−0.113	−0.043	−0.045	−0.314

Pearson’s correlation coefficient (r) analysis. * *p*-value < 0.05, ** *p*-value < 0.01. K-CPT: the Conners Kiddie Continuous Performance; yr.: year; GA: gestational age; wk.: week; BW: birth body weight; g: gram; PH: pulmonary hypertension; VSD: ventricular septum deficit; ASD: atrial septum deficit; PDA: patent ductus arteriosus; IVH: intraventricular hemorrhage; ROP: retinopathy of prematurity; RDS: respiratory distress syndrome; NEC: necrotizing enteritis; BPD: bronchopulmonary dysplasia; PVL: periventricular leukomalacia; BCVA: best corrected visual acuity; SPH: spherical correction; CYL: cylindricity; SE: spherical equivalent; RT: reaction time, SE: standard error, ISI: inter-stimulus interval. Note: Only visual data from the right eye (OD) were used. The VA is presented by logma values, with smaller values indicating better vision.

**Table 5:** The correlations between WPPSI-IV variables demographic data, clinical data, and vision.

	FSIQ	VCI	VSI	FRI	WMI	PSI	BD	IN	MR	BS	PM	SI	PC	CA	ZL	OA
Current age (yr.)	−0.048	−0.014	−0.037	0.076	−0.109	0.118	−0.044	0.046	0.002	0.165	−0.124 *	0.218 *	0.099	0.026	−0.053	0.02
Gender	0.11	0.062	0.150 *	0.067	0.133 *	−0.006	0.125 *	0.042	0.074	−0.006	0.081	0.135	0.119	0.194 *	0.161 *	0.204 **
Perinatal factors																
GA (wk.)	0.356 **	0.181 **	0.337 **	0.385 **	0.343 **	0.386 **	0.332 **	0.229 **	0.256 **	0.398 **	0.262 **	0.325 **	0.368 **	0.292 **	0.371 **	0.334 **
BW (g)	0.370 **	0.232 **	0.321 **	0.376 **	0.364 **	0.394 **	0.297 **	0.241 **	0.232 **	0.412 **	0.310 **	0.337 **	0.347 **	0.319 **	0.340 **	0.315 **
Apgar score, 1 min	0.302 **	0.157 *	0.245 **	0.305 **	0.279 **	0.255 **	0.253 **	0.153 *	0.211 **	0.323 **	0.237 **	0.211 *	0.323 **	0.211 *	0.263 **	0.275 **
Apgar score, 5 min	0.246 **	0.117	0.208 **	0.232 **	0.240 **	0.191 *	0.241 **	0.138 *	0.169 **	0.239 **	0.206 **	0.159	0.263 **	0.133	0.242 **	0.219 **
PH	−0.057	−0.069	−0.043	−0.046	−0.02	−0.106	−0.023	−0.099	−0.016	−0.085	−0.01	0.013	−0.074	−0.058	−0.04	−0.055
VSD	0.052	0.089	0.029	0.038	0.045	0.035	0.048	0.037	−0.014	−0.018	0.008	0.009	0.084	0.083	0.076	0.004
ASD	−0.075	−0.083	−0.054	−0.054	−0.088	0.035	0.003	−0.027	−0.011	0.002	−0.074	−0.075	−0.046	0.025	−0.08	−0.111
PDA	−0.225 **	−0.12	−0.214 **	−0.237 **	−0.256 **	−0.219 *	−0.208 **	−0.164 **	−0.142 *	−0.230 *	−0.167 **	−0.236 **	−0.257 **	−0.144	−0.287 **	−0.259 **
IVH	−0.173 **	−0.127 *	−0.195 **	−0.139	−0.185 **	−0.032	−0.200 **	−0.045	−0.131 *	0.013	−0.162 *	−0.088	−0.109	−0.097	−0.174 **	−0.218 **
ROP	−0.350 **	−0.171 **	−0.304 **	−0.465 **	−0.359 **	−0.366 **	−0.315 **	−0.181 **	−0.288 **	−0.421 **	−0.257 **	−0.313 **	−0.449 **	−0.367 **	−0.387 **	−0.299 **
RDS	−0.351 **	−0.196 **	−0.313 **	−0.394 **	−0.333 **	−0.378 **	−0.313 **	−0.211 **	−0.288 **	−0.439 **	−0.290 **	−0.278 **	−0.339 **	−0.308 **	−0.316 **	−0.280 **
NEC	−0.089	−0.055	−0.084	−0.223 *	−0.121	−0.12	−0.083	−0.078	−0.084	−0.240 **	−0.076	−0.104	−0.257 **	−0.184 *	−0.086	−0.066
BPD	−0.362 **	−0.175 **	−0.366 **	−0.386 **	−0.325 **	−0.402 **	−0.362 **	−0.239 **	−0.247 **	−0.394 **	−0.246 **	−0.380 **	−0.386 **	−0.306 **	−0.362 **	−0.359 **
Sepsis	−0.253 **	−0.180 **	−0.274 **	−0.296 **	−0.289 **	−0.222 *	−0.156 **	−0.189 **	−0.180 **	−0.188 *	−0.191 **	−0.305 **	−0.291 **	−0.291 **	−0.296 **	−0.295 **
Pneumonia	−0.316 **	−0.207 **	−0.311 **	−0.358 **	−0.287 **	−0.207 *	−0.321 **	−0.190 **	−0.205 **	−0.252 **	−0.194 **	−0.418 **	−0.312 **	−0.266 **	−0.328 **	−0.369 **
Anemia	−0.247 **	−0.169 **	−0.208 **	−0.209 *	−0.293 **	−0.228 *	−0.133 *	−0.168 **	−0.157 *	−0.283 **	−0.243 **	−0.229 **	−0.242 **	−0.200 *	−0.284 **	−0.221 **
Blood transfusion	−0.280 **	−0.199 **	−0.234 **	−0.290 **	−0.289 **	−0.291 **	−0.179 **	−0.196 **	−0.165 **	−0.329 **	−0.247 **	−0.273 **	−0.299 **	−0.276 **	−0.278 **	−0.243 **
Surfactant use	−0.315 **	−0.141 *	−0.319 **	−0.345 **	−0.297 **	−0.355 **	−0.339 **	−0.167 **	−0.213 **	−0.333 **	−0.221 **	−0.280 **	−0.387 **	−0.321 **	−0.327 **	−0.326 **
PVL	−0.062	−0.062	−0.123 *	0.06	−0.05	0.02	−0.106	−0.039	0.082	0.007	−0.042	−0.052	0.002	0.02	−0.04	−0.035
Vision																
BCVA	−0.169	−0.174	−0.104	−0.092	−0.009	−0.152	−0.137	−0.277 **	−0.058	−0.310 **	0.131	−0.230 *	−0.07	−0.097	−0.137	−0.055
SPH	−0.033	−0.08	0.044	0.039	0.058	−0.033	0.034	−0.025	−0.067	−0.004	0.071	−0.194	0.049	0.033	0.008	0.041
CYL	0.095	0.062	−0.105	0.057	0.158	−0.031	−0.001	0.136	0.083	0.117	0.03	0.00	−0.032	−0.17	0.237 *	−0.181
SE	0.086	0.04	−0.061	0.063	0.171	−0.029	0.031	0.137	0.041	0.12	0.058	−0.043	−0.012	−0.136	0.222	−0.13

Pearson’s correlation coefficient (r) analysis. * *p*-value < 0.05, ** *p*-value < 0.01. WPPSI: the Wechsler preschool and primary scale of intelligence; yr.: year; wk.: week; g: gram; GA: gestational age; BW: birth body weight; PH: pulmonary hypertension; VSD: ventricular septum deficit; ASD: atrial septum deficit; PDA: patent ductus arteriosus; IVH: intraventricular hemorrhage; ROP: retinopathy of prematurity; RDS: respiratory distress syndrome; NEC: necrotizing enteritis; BPD: bronchopulmonary dysplasia; PVL: periventricular leukomalacia; BCVA: best corrected visual acuity; SPH: spherical correction; CYL: cylindricity; SE: spherical equivalent; FSIQ: full scale IQ; VCI: verbal comprehension index; VSI: visual spatial index; FRI: fluid reasoning index; WMI: working memory index; PSI: processing speed index; BD: block design; IN: information; MR: matric reasoning; BS: bug search; PM: picture memory; SI: similarity; PC: picture concept; CA: cancellation; ZL: zoo locations; OA: object assembly. Note: Only visual data from the right eye (OD) were used. The VA is presented by logma values, with smaller values indicating better vision.

## Data Availability

The data that support the findings of this study are available from the corresponding author, Yu-Shu Huang, upon reasonable request.
